# The Virtual Trial

**DOI:** 10.3389/fnins.2017.00110

**Published:** 2017-03-07

**Authors:** Willem de Haan

**Affiliations:** Department of Neurology, VU University Medical CenterAmsterdam, Netherlands

**Keywords:** connectivity, network, Alzheimer, neurodegeneration, computational modeling, graph theory

## Abstract

Although brain network analysis in neurodegenerative disease is still a fairly young discipline, expectations are high. The robust theoretical basis, the straightforward detection and explanation of otherwise intangible complex system phenomena, and the correlations of network features with pathology and cognitive status are qualities that show the potential power of this new instrument. We expect “connectomics” to eventually better explain and predict that essential but still poorly understood aspect of dementia: the relation between pathology and cognitive symptoms. But at this point, our newly acquired knowledge has not yet translated into practical methods or applications in the medical field, and most doctors regard brain connectivity analysis as a wonderful but exotic research niche that is too technical and abstract to benefit patients directly. This article aims to provide a personal perspective on how brain connectivity research may get closer to obtaining a clinical role. I will argue that network intervention modeling, which unites the strengths of network analysis and computational modeling, is a great candidate for this purpose, as it can offer an attractive test environment in which positive and negative influences on network integrity can be explored, with the ultimate aim to find effective countermeasures against neurodegenerative network damage. The virtual trial approach might become what both dementia and connectivity researchers have been waiting for: a versatile tool that turns our growing connectome knowledge into clinical predictions.

## Introduction

Neuronal connectivity as explicatory model of brain function has been around for quite some time, and it has offered valuable perspectives, but has never reached the status of inevitable clinical tool. The classical “disconnection syndromes” as proposed by legends like Wernicke and Dejerine elegantly explained neurological symptoms (Catani and ffytche, 2005; Catani and Mesulam, 2008), but have had a hard time surviving the neophrenological dominance in the neurological practice of the past decades, where advancing imaging techniques and lesion studies have fueled our intrinsic tendency to localize every (dys)function of the brain, even though we know that particularly our cognitive abilities are heavily dependent on distributed circuits.

But connectivity is making a comeback. The convergence of technical advances in brain imaging, and improved signal analysis methods for non-linear brain dynamics enables a new perspective on the connectivity and communication between brain regions. On top of that, major breakthroughs in the field of complex network analysis in the late nineties, introduced novel ways to understand brain connectivity: it is not just a matter of “what is connected to what,” but it is the insight that connection patterns themselves can actually determine network function to a large extent. The power of modern complex network analysis is the manner in which it focuses on the meaningful core of complex systems, and offers powerful yet easy to grasp insights that can be quantified with a large arsenal of measures (Rubinov and Sporns, 2010; Fornito et al., 2015). This has offered not just theoretical but crucial practical solutions in a wide range of fields, such as infrastructure, economy, telecommunication, social science, and more (Newman, 2003; Boccaletti et al., 2006; Mitchell, 2006). In humans and other animals, studies have convincingly demonstrated that structural and functional brain networks indeed possess smart organizational principles, and, regarding neurodegenerative disease, that the link between connectivity, pathology, physiology of the brain, and cognition is real, suggesting connectomics to be a powerful new tool to understand and perhaps predict disease (Pievani et al., 2011; Tijms et al., 2013; Stam, 2014).

The enthusiasm of the connectivity research community is met with skepticism from more clinically oriented peers, who point to the fact that all the theoretical beauty and power has not yet cured a single person with dementia, or any other disease. The clinician who faces patients with cognitive decline and has practically nothing to offer but empathy, care and a few symptomatic drugs, will shrug his shoulders when confronted with the latest correlation between network hubs and amyloid plaques. Are we too optimistic? Well, probably it is too early to judge, and naïve to expect that a complex system like the brain will reveal its fundamental principles so quickly, when many technical and methodological hurdles have to be taken, and there are still so many unknown routes to explore.

Can we envision a more practical application of brain connectivity research to the clinical domain of neurodegenerative disease? One potential use is the development of diagnostic and prognostic biomarkers. However, in this paper I will take a different, more ambitious stance and argue that connectomics may even directly contribute to the development of new treatments for dementia. To clarify this idea, several stages can be recognized. First, I will briefly summarize the results of brain network analysis in dementia; how do we get from neurodegenerative disease data to a “connectopathy.” Subsequently, we move to a phase in which the observed network damage is incorporated into a dynamic, virtual disease simulation; from connectopathy to computational neurodegenerative network model.

The final stage and main focus of this paper, the “virtual trial,” deals with the challenge to predict successful interventions by testing strategies that restore network integrity in the model, based on the assumption that this will be beneficial for cognitive performance. A practical example of this approach will be demonstrated, backed by previous publications and current research projects, and taking into consideration potential pitfalls and improvements.

## From neurodegenerative disease to connectopathy

Early reports of connectivity analysis applied to dementia already date back several decades (De Lacoste et al., 1984; Leuchter et al., 1992). As expected, the loss of neurons and synapses results in an overall breakdown of connectivity, prompting authors to label its most common form, Alzheimer's disease (AD) as a “disconnection syndrome” (Delbeuck et al., 2003). The notion that the gradual disconnection between brain regions is the cause of the cognitive deterioration is interesting, but it is not very specific. Fortunately, several breakthroughs in complex network theory in the late 90's caused an explosive growth of interest in complex system analysis: now there were new tools to understand connectivity patterns, not just describe them (Watts and Strogatz, 1998; Barabasi and Albert, 1999; Strogatz, 2001; Newman, 2003; Boccaletti et al., 2006). Complex network analysis applied to human brain data started slightly over a decade ago, with the first evidence of small-world network topology in human resting-state magneto-encephalography (MEG) data (Stam, 2004). This illustration of efficient network topology in the human brain has since then been demonstrated in many studies (Sporns and Zwi, 2004; Bassett and Bullmore, 2006; He et al., 2007; Kwok et al., 2007; Stam et al., 2007; van den Heuvel et al., 2008; Stam, 2010). Furthermore, the repeated finding that in neurodegenerative disease this structure is gradually lost sparked the quest for further determination of network damage patterns in structural and functional networks on different scales (Supekar et al., 2008; Bassett and Bullmore, 2009; de Haan et al., 2009; Sanz-Arigita et al., 2010; Pievani et al., 2011; Xie and He, 2011; Stam, 2014). With network theory, we could start to explain *why* the observed brain changes were bad for its function.

Soon, other fascinating findings were reported: the striking overlap between patterns of amyloid pathology and the presence of highly connected areas (hubs), as reported by Buckner et al. (2009), or different types of dementia producing different network damage patterns (de Haan et al., 2009; Seeley et al., 2009; Zhou et al., 2010). Since then, neurodegenerative disease (and particularly AD) has been a frequent focus of network research. The combined results so far offer the following picture: “connectopathy” occurs at an early stage, progresses gradually, is fairly dementia-specific, and correlates with disease severity and pathology (Pievani et al., 2011; Tijms et al., 2013; Stam, 2014). It is therefore reasonable to acknowledge the potential of this type of analysis. Of course, since brain network research is still a very young field, the reliability and reproducibility of many results has to be confirmed. There is a lot of discussion about network measure definition, applicability of graph theoretical analysis to brain networks of limited size, methods to compare different networks in an unbiased way, network-specific statistical problems, and more (Deuker et al., 2009; van Wijk et al., 2010; Zalesky et al., 2010; Wang et al., 2011).

In search of a practical use of this knowledge, biomarker development is an obvious next step. At present however, the sensitivity and specificity of network and connectivity-related measures as diagnostic markers do not seem to perform better than more commonly known structural or functional measures, like atrophy rate, cerebral spinal fluid (CSF) protein levels or oscillatory slowing (Damoiseaux and Greicius, 2009; He et al., 2009; Koch et al., 2012; Gomez-Ramirez and Wu, 2014; Fornito et al., 2015). Similarly, the use of these markers to monitor or predict disease course has not been demonstrated. Perhaps, combinations of markers may improve their accuracy (Poil et al., 2013; Dauwan et al., 2016; Khazaee et al., 2016).

Brain network analysis in dementia may not tell the whole story, but at least it seems capable of examining a poorly understood and (perhaps therefore) underestimated aspect of dementia. And, with a steady stream of scientific studies, gradually producing a more nuanced view of longitudinal changes in both structural and functional connectivity patterns in dementia, we can now start to ask ourselves the following questions: can we take advantage of this abstract realm of network analysis, integrate damage features into an explicatory model, and find general principles of damage that can point us toward the core of the disease mechanism, and possible targets for future interventions?

## From connectopathy to neurodegenerative network model

Ideally, from the combined findings of brain connectivity studies in neurodegenerative disease a clear and consistent picture of disease-specific damage should emerge. However, since there are different types of dementia, different modalities, different stages of disease severity, and different hypotheses, bringing together all the evidence is not an easy task. Moreover, underlying patterns, mechanisms and causal relations in complex network data may be completely invisible to the naked eye. Therefore, interpretation of network damage should be backed up by appropriate analysis. One way to do this is by a digital variant of lesion studies: simulating brain network damage. By mimicking different types of damage in a brain network model, one can test if certain “damage principles” apply: what is the minimal assumption that we have to make to replicate AD network damage? These “virtual lesions” can be focal, global, structural and/or dynamic (Kaiser et al., 2007; Honey and Sporns, 2008; Alstott et al., 2009; Stam et al., 2010), resembling focal vs. more global neurological pathology. For example, to characterize the type of network damage in AD, a resting-state MEG study compared two different damage types: “targeted attack,” which selectively weakened hub regions, and “random error,” which applied random network damage (Stam et al., 2009). Since the first damage algorithm turned out to resemble the patient data more closely, this appeared to confirm the earlier described hub vulnerability in AD. Somehow, highly connected regions were prone to AD.

**Figure 1:**
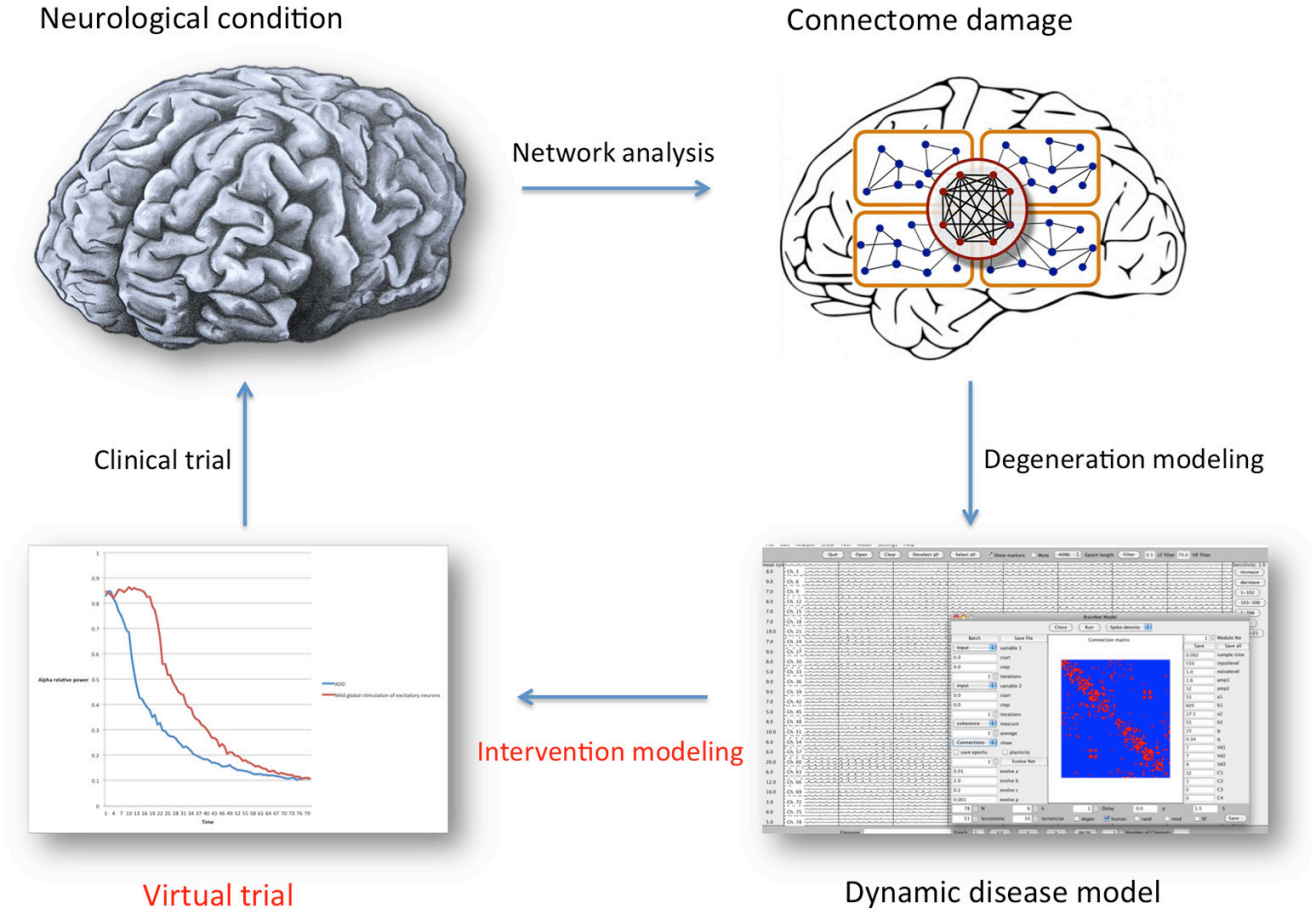
**General overview of the brain network analysis and computational modeling process described in this article, with the special focus indicated in red text**. Once a representative network disease model has been constructed, many properties (regarding neuronal behavior, functional or structural connectivity) can be altered as a way to introduce a defense mechanism: intervention modeling. The underlying assumption is that retaining “normal” network organization is beneficial. Intervention scenarios can then be compared statistically, and serve as a guide for future clinical trial design. For this purpose, model validation and translation to specific clinical treatment options presents a considerable challenge.

To investigate this intriguing finding further, in a recent modeling study we hypothesized that the reason for this was the high mean levels of activity in hubs, since neuronal hyperactivity and -excitability are increasingly reported in early AD stages (Doble, 1999; Celone et al., 2006; Dickerson and Sperling, 2009; Palop and Mucke, 2009, 2010; Santos et al., 2010; de Haan et al., 2012; Mehta et al., 2013; Busche and Konnerth, 2015; Busche et al., 2015; Oh et al., 2015; Yuan and Grutzendler, 2016). For this purpose, we used a dynamic model with neural masses coupled according to human large-scale topology, to be able to simulate global network function while altering neuronal excitability (see Figure 1). Under the assumption of activity dependent damage, we reproduced all major AD functional network hallmarks (including initial hyperactivity and hyperconnectivity in the early stage, as has been reported in MCI patients; Pijnenburg et al., 2004; Celone et al., 2006; van Deursen et al., 2008; Maestu et al., 2011; Maestú et al., 2015), supporting the view that neuronal hyperactivity may play more than just a supporting role in the disease process itself. Whether this particular study truly reveals a pathophysiological principle of AD remains to be confirmed, but here it mainly serves to illustrate the potential of network modeling.

When investigating brain network lesions and disease mechanisms, modeling should not be regarded as an interesting side step to real experiments; it is indispensable, because disturbance of complex, dynamical, non-linear systems never produces strictly local, simple effects, and examination or prediction of cause-effect relations is therefore almost impossible without it. By incorporating relevant variables in a computational model, which produces output that can be directly compared to and analyzed with the same network analysis tools as patient data, a much more reliable grasp on system-wide effects can be achieved than by making educated guesses (Raj, 2015). The flexibility of network modeling means it can be tailored to specific hypotheses or questions, but it can also be a pitfall: with endless parameters to adjust, anatomical details to add and neuronal behavior to incorporate, one can easily “get lost in modelspace” and never return. This is why clinicians are important: realistic biological questions, hypotheses and observations are needed to feed into and constrain these models.

## From neurodegenerative network model towards therapy: the virtual trial

If network modeling is an appropriate tool to simulate neurodegenerative disease, why not reverse our thinking, and test potentially positive influences, to learn how to preserve adequate connectivity and slow down or even reverse dementia? Can we implement a general principle to make the network react differently, for example by adjusting oscillatory properties? Can we make the network survive? In our example, where we coupled neural mass models according to human topology to simulate a dynamic human large-scale network (that produces EEG-like output), we can alter the excitability of neurons and see the resulting network behavior. This way, the intervention mechanism of medication or other ways of stimulation/inhibition can be simulated: for example, we can inhibit excitatory neurons in the case of a hyperactive network that collapses due to hyperexcitation.

**Figure 2:**
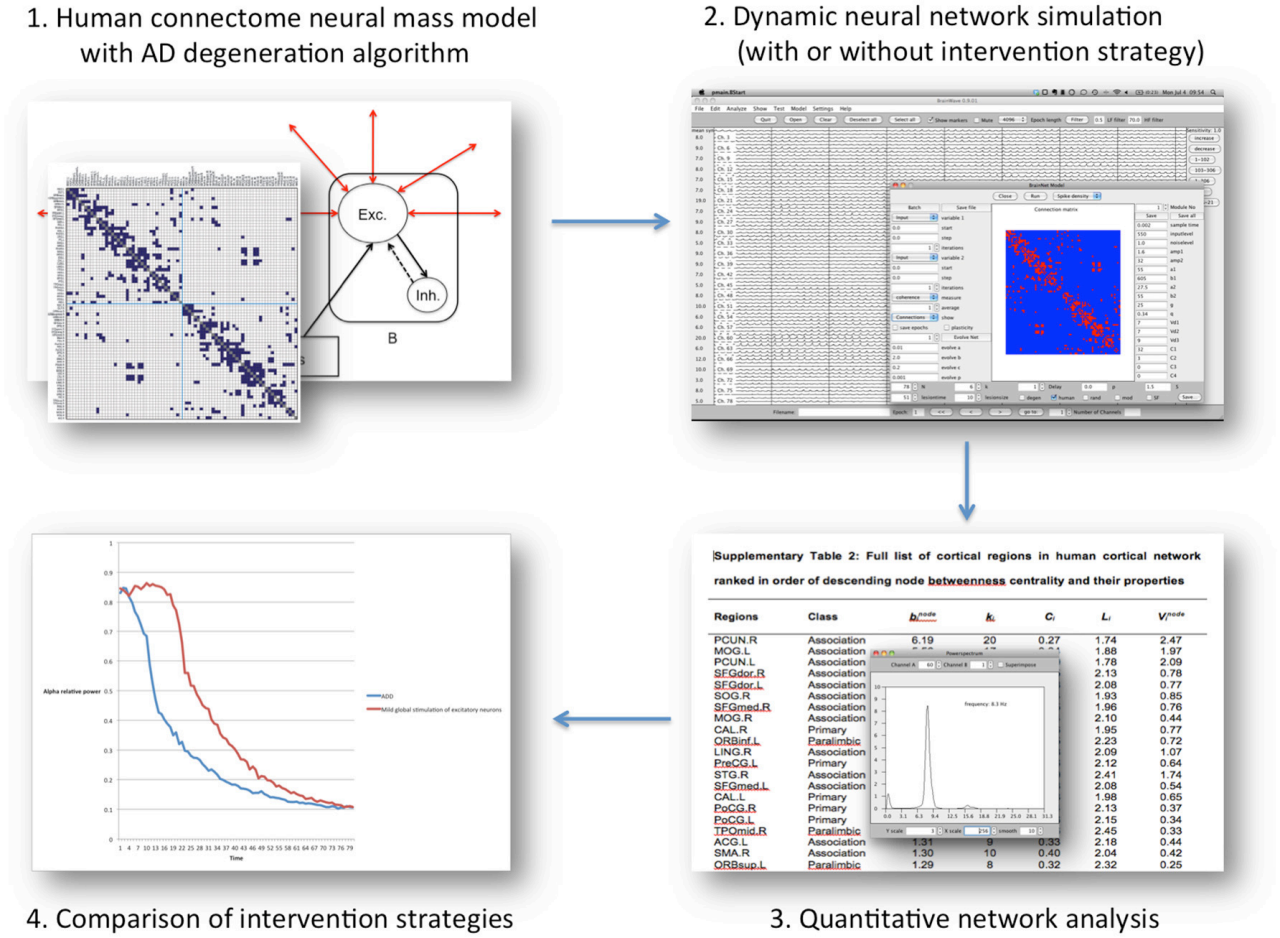
**Overview of the “Virtual Trial” procedure in the computational model mentioned in the text**. Regional neural dynamics are based on 78 neural mass models, and are connected according to human, DTI-derived topology. The resulting dynamic network generates EEG-like data, which can be analyzed in the same way as patient data. During the simulation negative (degeneration algorithm) and positive influences (intervention scnearios) can be introduced by altering neuronal behavior characteristics or connectivity. The subsequent effect on the network level over time can be assessed, and quantitatively compared between scenarios.

This is precisely the strategy we adopted when trying to restore network properties in our AD simulation (de Haan ea, under review, see Figure 2). We compared it with 5 other strategies, in which inhibitory and excitatory neurons were either stimulated or inhibited simultaneously or selectively. We ran these strategies and tracked network status by assessing commonly used parameters to describe functional and structural connectivity, network topology, hub status and oscillatory behavior. Although global inhibition appeared to be a fairly successful strategy, to our surprise the best way to defend the network was by stimulating excitatory neurons. Apparently, on a network level this strategy shifts the excitation-inhibition balance in a positive way; the intervention more than doubled the time before network collapse, independent of intervention starting delay, which can be considered a success.

Of course, this exploratory study may still suffer from methodological limitations. For example, we did not include any plasticity effects, which may have underestimated the potential of the interventions. Or we could have added a greater level of detail (more neural masses, anatomical landmarks). Still, it does demonstrate a few noteworthy things: first, subtle small-scale adjustments (that don't cause notable changes in “healthy” network simulations) can profoundly influence the large-scale network reaction to degeneration; with the right strategy, we may be able to counter pathological processes in an elegant way. Second, the counter-intuitive success of stimulation in a hyperactive network underscores the complex behavior of non-linear systems, and thus the need for modeling.

Network intervention modeling is not limited to the neurophysiological domain. Any model that incorporates a fairly plausible degeneration process and defense mechanism can be used for the same purpose. For example, there could be an algorithm describing amyloid deposition, spreading in a spatiotemporal pattern as observed in clinical studies, and exerting a toxic effect on its surroundings. Then, an amyloid clearance intervention may be introduced to test network preservation. Although it may take time to develop adequate and insightful models, attacking the AD mystery with both top-down (clinical studies) and bottom-up (modeling) approaches that hopefully inspire each other may speed up our understanding of the disease.

Ultimately, successful virtual interventions should be translated into actual clinical treatments, and there are many ways to manipulate brain networks (medication, stimulation, surgery). However, the major challenge is to determine the optimal strategy, and network intervention modeling is a rational intermediate step that may guide trial design. Since intervention modeling will, by definition, not be perfect, the clinical findings may differ and be less persuasive. Here, in-trial monitoring of network changes may inform us of where practice and theory grow apart, and how to update our insights, and our model. Another step that might be included is testing modeling predictions in AD mouse models, since faster simulation-trial cycles can be realized than in human trials.

## Conclusion

The merit of brain connectivity research goes beyond the elucidation of normal and pathological brain organization, or the development of diagnostic or prognostic markers. Network intervention modeling can offer a versatile test environment in which positive and negative influences on network integrity can be explored, with the aim to counter neurodegenerative network damage.

By testing and directly comparing relevant and realistic clinical hypotheses, the virtual trial approach could become a fast, flexible and inexpensive method to bring our rapidly growing knowledge of brain connectivity to the patient, and to provide the dementia research community with a highly needed new perspective and a tool that may boost the success rate of the inevitably slow, expensive and invasive clinical trials.

If we truly accept the view of our brain as a dynamic, complex, decentralized system from which cognitive traits emerge, and connectivity analysis as the tool with the firmest grip on this aspect of the brain, it is time to move on from educated guessing to prediction modeling, when it comes to intervening. This route from byte to bedside may unite many different types of technically and clinically oriented neuroscientists.

## Author contributions

The author confirms being the sole contributor of this work and approved it for publication.

### Conflict of interest statement

The author declares that the research was conducted in the absence of any commercial or financial relationships that could be construed as a potential conflict of interest.
